# Can non-participants in a follow-up be used to draw conclusions about incidences and prevalences in the full population invited at baseline? An investigation based on the Swedish MDC cohort

**DOI:** 10.1186/s12874-023-02053-w

**Published:** 2023-10-11

**Authors:** Anton Nilsson, Jonas Björk, Ulf Strömberg, Carl Bonander

**Affiliations:** 1 Epidemiology, Population Studies and Infrastructures (EPI@LUND), Tornblad Institute, Lund University, Biskopsgatan 9, Hämtställe 21, 22362 Lund, Sweden; 2 Clinical Studies Sweden, Forum South, Skåne University Hospital, Lund, Sweden; 3 Health Economics and Policy, School of Public Health & Community Medicine, Institute of Medicine, University of Gothenburg, Gothenburg, Sweden; 4 Centre for Societal Risk Research, Karlstad University, Karlstad, Sweden

**Keywords:** Generalizability, Representativity, Self-selection, Mortality, Risk factors, Continuum of resistance

## Abstract

**Background:**

Participants in epidemiological cohorts may not be representative of the full invited population, limiting the generalizability of prevalence and incidence estimates. We propose that this problem can be remedied by exploiting data on baseline participants who refused to participate in a re-examination, as such participants may be more similar to baseline non-participants than what baseline participants who agree to participate in the re-examination are.

**Methods:**

We compared background characteristics, mortality, and disease incidences across the full population invited to the Malmö Diet and Cancer (MDC) study, the baseline participants, the baseline non-participants, the baseline participants who participated in a re-examination, and the baseline participants who did not participate in the re-examination. We then considered two models for estimating characteristics and outcomes in the full population: one (“the substitution model”) assuming that the baseline non-participants were similar to the baseline participants who refused to participate in the re-examination, and one (“the extrapolation model”) assuming that differences between the full group of baseline participants and the baseline participants who participated in the re-examination could be extended to infer results in the full population. Finally, we compared prevalences of baseline risk factors including smoking, risky drinking, overweight, and obesity across baseline participants, baseline participants who participated in the re-examination, and baseline participants who did not participate in the re-examination, and used the above models to estimate the prevalences of these factors in the full invited population.

**Results:**

Compared to baseline non-participants, baseline participants were less likely to be immigrants, had higher socioeconomic status, and lower mortality and disease incidences. Baseline participants not participating in the re-examination generally resembled the full population. The extrapolation model often generated characteristics and incidences even more similar to the full population. The prevalences of risk factors, particularly smoking, were estimated to be substantially higher in the full population than among the baseline participants.

**Conclusions:**

Participants in epidemiological cohorts such as the MDC study are unlikely to be representative of the full invited population. Exploiting data on baseline participants who did not participate in a re-examination can be a simple and useful way to improve the generalizability of prevalence and incidence estimates.

**Supplementary Information:**

The online version contains supplementary material available at 10.1186/s12874-023-02053-w.

## Introduction

Participants in cohort studies are rarely fully representative of the target populations that researchers and policymakers wish to make inferences about. In an often-cited review of selective participation in epidemiologic studies, Galea and Tracy [1] noted that study participation tends to vary with respect to socio-demographic characteristics and health-related behaviors, where in particular males, persons of lower socioeconomic status, and persons with an unhealthy lifestyle are less likely to participate. Such lack of representativeness potentially limits the generalizability of sample-based estimates of prevalences, as well as incidences and other outcomes in the follow-up, as these may be influenced by factors that the study is unrepresentative with respect to [2].

Conventional approaches to tackle lack of generalizability include direct standardization and inverse probability of participation weighting [3–6]. These methods work by “balancing” the participant sample with respect to a set of characteristics that are observed both in the sample and in the target population. With direct standardization, for example, mean outcomes are calculated within each stratum, defined by different combinations of characteristics. One then determines a weighted sum of the strata-specific means, where the weights given by the shares of the individuals in the population who belong to the different strata. Inverse probability of participation weighting is non-parametrically equivalent to the above procedure, but instead of reweighting the data based on the probability of each combination of characteristics, this method reweights the data based on the probability of being part of the sample, given the individual’s combination of characteristics. The advantage of this approach is that it can be implemented parametrically, allowing for combinations of characteristics with few or no individuals present in the sample.

In practice, many determinants of study participation may not be observable both in the participants and in the target population of interest, meaning that methods such as direct standardization and inverse probability of participation weighting are not necessarily helpful. We have, for example, demonstrated that reweighing the baseline participants in the Swedish Malmö Diet and Cancer (MDC) cohort study with respect to socio-demographic factors and previous hospital admissions helped only little to bring mortality rates among the participants closer to the higher ones observed in the full invited population [7].

In studies where recruitment involves one or several reminders, the *continuum of resistance model* [8–10] has sometimes been used as an alternative approach to try to improve the generalizability of prevalence and incidence estimates. According to the model, individuals who agreed to participate in a study only after receiving reminders are more resemblant of non-participants than what study participants who agreed to participate already after the first invitation are. As a result, data on those who only participated after receiving reminders may be exploited to generalize study results to the population. This may be done, for example, by applying a substitution model where non-participants are assumed to be similar to late participants [11–14], or an extrapolation model [8, 9, 14] where differences between early participants and the full group of participants are assumed to be informative about differences between the full group of participants and the full population. Many studies, mostly based on surveys, have provided evidence in favor of the continuum of resistance model [11–22], as late participants were found to deviate from early participants in ways that were at least qualitatively similar to how non-participants are known to differ from participants. Other studies, however, have found little or no evidence of a continuum [23–26].

In this work, we propose another variant of the continuum of resistance model. Instead of exploiting participation after reminders as an indication of the willingness to participate, we suggest that one may exploit data on individuals who participated at baseline but refused to participate in a follow-up examination. Arguably, such individuals have a lower propensity for participation than what baseline participants in general have, and they may therefore be more similar to baseline non-participants. In turn, data on these individuals may be exploited to draw inferences about the full invited population. Assessing this idea, we used data from the MDC study to compare disease incidences, mortality, and prevalences in the full group of baseline participants with the group of baseline participants who did not participate in a five-year re-examination. We also compared these groups with the full invited population as well as with the invited ones who did not participate at baseline. Further, we examined the ability of two empirical methods based on the continuum of resistance model to reproduce results in the full invited population without using data on baseline non-participants. We focused on prevalences, disease incidences, and mortality as such, rather than on associations between these and other factors, as previous research based on MDC and other cohorts has suggested that associations tend to be highly generalizable, even if the participants are not representative [7, 27–29]. Nevertheless, prevalences, incidences, and mortality are of interest in themselves, as they paint a picture of the health and other features of the population, providing useful descriptive information and clues as to what health outcomes, health-related behaviors, or circumstances may potentially be improved through intervention.

## Methods

### Data

The MDC study is a cohort study conducted in the city of Malmö, southern Sweden. Recruitment took place between 1991 and 1996, with a participation rate of approximately 40%. At baseline, participants filled out a questionnaire about health, diet, and lifestyle, such as smoking and drinking behavior. There were also measurements of blood pressure, body composition, and anthropometric measures, such as height and weight. Baseline participants were later invited to a five-year re-examination, where they filled out a similar questionnaire. The re-examination was completed in August 2001.

In Sweden, researchers with an ethical approval can apply to access pseudonymized data from administrative population registers. Many of these registers, such as the *Total Population Register* and registers of education levels and incomes, are maintained by Statistics Sweden, a government agency that is responsible for collecting official statistics and from which researchers routinely obtain data extracts. Statistics Sweden can also link data from their own registers to registers maintained by other agencies, such as the National Board of Health and Welfare, or to research data that has been submitted by investigators.

Our data were delivered by Statistics Sweden and came in two separate sets: one comprising the full background population and one comprising those who participated in the MDC study at baseline. While both these datasets were linked to several sources by Statistics Sweden, there were no links between the two datasets, meaning that individuals participating in MDC could not be directly identified in the dataset comprising the full background population. The background population, which essentially corresponded to those invited to the MDC study, consisted of all males (born 1923–1945) and females (born 1923–1950) who lived in Malmö at some point between January 1, 1991, and September 30, 1996, a population of 74,103 individuals. In practice, some individuals in the background population where not invited because of death, migration, or for other reasons [30]. There were 28,096 individuals who completed all the baseline examinations. Of these, 22,366 participated in the five-year re-examination. The dataset comprising baseline participants contained information on whether individuals also participated in the re-examination.

Participation in the re-examination is assumed to depend on a person’s willingness to participate in the study. In addition, persons who had died prior to the re-examination would clearly not be able to participate in it, and the same would typically be the case for individuals living abroad. Differences between participants and non-participants in the re-examination may thus not only represent differences in the propensity for participation but also differences in the propensities to die or migrate, processes that are likely to be driven by other factors than the propensity to participate in a study. To prevent deaths and migrations from influencing our results, we limited attention to individuals who had not died or emigrated before September 1, 2001.

Our datasets included administrative data from Statistics Sweden on socio-demographics in 1990–2000. These included year of birth, sex, civil status, country of birth (grouped), migration events, education, and income. Moreover, the datasets were linked to registers from the *National Board of Health and Welfare*, including the Patient Register and the Cause of Death Register. The Patient Register covered all diagnoses (coded according to ICD-9/ICD-10) and dates of inpatient hospital visits in the period 1987–2016 as well as all outpatient hospital visits in 2001–2016. The Cause of Death Register included all deaths in the period 1991–2016, with data on the date of the death and causes of death. To ensure high data quality, we only made use of primary diagnoses and primary causes of death.

To measure individual characteristics up to the re-examination, we used data on socio-demographics in 2000, or when this was not available (if the individual lived abroad in 2000) in the latest year available. To measure disease history, we exploited data on hospitalizations between 1987 and August 2001. Six categories of hospitalizations were created, based on recorded diagnoses: neoplasms (ICD codes 140–239/C00-D48), diabetes (250/E10-E14), mental and behavioral disorders (290–319/F00-F99), diseases of the circulatory system (390–459/I00-I99), diseases of the respiratory system (460–519/J00-J99), and diseases of the digestive system (520–579/K00-K93). We created binary indicators for whether an individual had had at least one hospitalization for each of these six categories.

As for outcomes in the follow-up (after the re-examinations were completed), we examined all-cause mortality and both mortality and incidence of cardiovascular disease (CVD), cancer, smoking-related conditions, and alcohol-related conditions. CVD mortality was defined by ICD-10 codes I00–99 and incident CVD was defined by the occurrence of either a coronary event (a fatal or nonfatal myocardial infarction, I21, or a death due to ischemic heart disease, I22/I23/I25) or a fatal or nonfatal stroke (I60/I61/I63/I64), whichever came first. Cancer outcomes were defined based on ICD codes C00–99. Codes used to define smoking-related and alcohol-related conditions are provided in Table S1. Individuals who had not experienced the outcome under consideration before September 1, 2001, were followed from this date and until the first event under consideration occurred, until death or migration, or at most until the end of 2016, yielding follow-up times up to 15.33 years (mean 12.68 in the full population, 13.25 among the baseline participants, and 13.33 among the baseline participants who also participated in the re-examination).

We also used data on smoking behavior, risky drinking, and overweight and obesity from the baseline examination. For smoking, we used the four self-reported categories yes, regularly; yes, occasionally; no, stopped; and no, never. For drinking, we used self-reported data on frequency and intensity of the consumption of different alcoholic beverages, which were converted into average grams of alcohol intake per day. We then created an indicator for risky drinking equaling 1 if intake was more than 40 g per day in males or more than 20 g per day in females [31]. Overweight was defined as 25 ≤ BMI < 30 and obesity as BMI ≥ 30 [32].

### Data analysis

We compared the distributions of socio-demographics, disease history, and post-re-examination mortality and disease incidence rates across the background population, baseline participants, baseline participants who participated in the five-year re-examination, and baseline participants who did not participate in the five-year re-examination. Comparisons with the full population were summarized by calculating the Mean Squared Error (MSE), the average of the squared differences between the prevalence of each characteristic or incidence of each outcome in a given group and the corresponding values in the full background population. We calculated one MSE based on prevalences of the selected socio-demographic characteristics and disease history, and another one based on mortality and disease incidences. We also compared the characteristics and outcomes in the different groups with the corresponding numbers among individuals who did not participate in the baseline examination. While this group could not be directly observed, we identified their distributions and incidence rates by combining information from the full population sample and the participant sample and backing out the relevant numbers.

To examine the usefulness of the continuum of resistance model to reconstruct information on the background population, we applied a substitution and an extrapolation approach. In the substitution approach [11–14], we replaced all prevalences, outcome proportions, and average follow-up times in the group not participating at baseline by the corresponding information in the group participating at baseline but not in the re-examination. We then re-calculated the quantities of interest for the background population.

Specifically, let $\overline{y }$ denote a prevalence, an outcome proportion, or an average follow-up time in the full background population. By construction, we can write $\overline{y }$ as:

1 $$\overline{y }={\omega }_{r}\overline{{y }_{r}}+{\omega }_{b\backslash r}\overline{{y }_{b\backslash r}}+(1-{\omega }_{r}-{\omega }_{b\backslash r})\overline{{y }_{n}}$$

Here, ${\omega }_{r}$ is the proportion of the population participating in the re-examination, ${\omega }_{b\backslash r}$ the proportion of the population participating at baseline but not in the re-examination, and$\overline{{y }_{r}}$,$\overline{{y }_{b\backslash r}}$, $\overline{{y }_{n}}$ denote the relevant quantity among individuals participating in the re-examination, individuals participating at baseline but not in the re-examination, and individuals not participating at baseline. The substitution approach relies on the assumption that $\overline{{y }_{n}}=\overline{{y }_{b\backslash r}}$. Hence, to evaluate the usefulness of this method, we replaced $\overline{{y }_{n}}$ by $\overline{{y }_{b\backslash r}}$ in Eq. (1) and calculated $\overline{y }$ under this assumption.

In the extrapolation approach [8, 9], we instead assumed a linear relationship between participation propensity and the observed prevalence or incidence rate. Specifically, for each characteristic or outcome under consideration, we determined a linear equation:

2 $$\overline{y }=\alpha +\beta x$$

The parameters $\alpha$ and $\beta$ were determined such that the equation would fit the observations $({x}_{r},\overline{{y }_{r}})$ and $({x}_{b},\overline{{y }_{b}})$, where ${x}_{r}$ was the share of the background population that participated in the re-examination, $\overline{{y }_{r}}$ the observed prevalence or incidence rate in this group, ${x}_{b}$ the share of the background population who participated at baseline regardless of whether they participated in the re-examination, and $\overline{{y }_{b}}$ the observed prevalence or incidence rate in this group. Considering the interval of $x$-values from 0 to 1, the linear equation is assumed to measure the hypothetical observed prevalence or incidence rate in monotonically increasing subsets of the population, where increasingly reluctant participants are being included. To estimate characteristics and outcomes to the full background population, we thus set $x=1$ and determined the $\overline{y }$-value according to the line.

We also constructed Kaplan–Meier (KM) survival curves to graphically compare mortality and disease incidence across the different groups of individuals, as well as displaying the corresponding results calculated according to the substitution and extrapolation approaches.

Finally, we compared smoking, drinking, and body mass across baseline participants, re-examination participants, and re-examination non-participants, and examined the consequences of applying the substitution and extrapolation approaches, aiming to reconstruct the true (but unobservable) values in the full background population.

Statistical analyses were conducted in Stata version 16.1 (StataCorp).

## Results

**Fig. 1:**
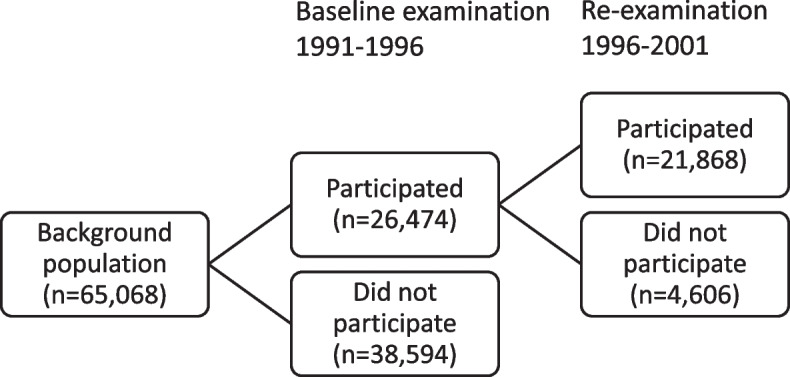
The different groups of participants and non-participants considered

Excluding individuals who had died or emigrated before September 1, 2001, the background population consisted of 65,068 individuals, of which 26,474 were baseline participants and 38,594 were baseline non-participants (Fig. 1). Of the baseline participants, 21,868 also participated in the re-examination, whereas 4,606 did not (Fig. 1). In Table 1, we report distributions of background characteristics across the groups. Baseline participants tended to be older than baseline non-participants, and were less likely to be male, more likely to be born in Sweden, more likely be married, and more likely to have higher socioeconomic status. They were also less likely to have a history of mental disease. Contrasting the percentages for different characteristics across baseline participants and the full background population, an MSE of 10.7 was obtained.

**Table 1:** Background characteristics (%)

	Background population (*n* = 65,068)	Baseline non-participants (*n* = 38,594)	Baseline participants (*n* = 26,474)	Baseline participants, participating in re-examination (*n* = 21,868)	Baseline participants, not participating in re-examination (*n* = 4,606)	Substitution approach to reconstruct background population	Extrapolation approach to reconstruct background population
*Socio-demographics*							
Age							
50–59	36	38	33	31	39	36	43
60–69	37	36	37	37	36	36	35
70–77	28	26	30	31	26	27	22
Legal sex							
Male	40	41	38	38	38	38	38
Country of birth							
Sweden	79	73	88	89	83	85	80
Civil status							
Married	55	52	61	62	55	57	50
Education							
Primary	42	46	36	35	38	37	39
Short secondary	28	26	30	30	30	30	30
Long secondary	11	10	13	13	12	12	12
Tertiary	19	17	22	22	20	21	19
Employment status							
Employed	33	31	36	36	37	37	38
Unemployed	8.6	10	5.8	5.6	7.0	6.5	7.9
Sickness absence	3.5	3.9	2.8	2.5	4.0	3.5	4.9
Retired	54	54	55	56	52	53	50
Disposable income							
Quintile 1	20	23	16	16	17	16	17
Quintile 2	20	22	17	17	19	18	20
Quintile 3	20	20	20	20	21	20	21
Quintile 4	20	18	23	24	22	23	21
Quintile 5	20	17	24	25	22	23	20
*Disease history*							
Circulatory	16	17	16	16	17	16	18
Diabetes	1.8	2.2	1.2	1.2	1.5	1.4	1.7
Neoplasms	10	9.6	11	11	11	11	11
Respiratory	5.7	6.1	5.1	5.0	5.9	5.6	6.5
Digestive	13	12	13	13	15	14	16
Mental	5.4	7.0	3.1	2.6	5.4	4.5	7.2
MSE			10.7	13.6	4.4	5.4	8.4

Socio-demographics refer to December 31, 2000. Disease history refers to whether the individual was hospitalized for the disease type in question between January 1987 and August 2001. Throughout, individuals who had died or did not live in Sweden by September 1, 2001, were excluded. MSE refers to mean squared error, calculated across all prevalences in the table, in a comparison with the background population

Among the baseline participants, those who did not participate in the re-examination were generally much more similar to the baseline non-participants. Overall, the characteristics of the baseline participants who did not participate in the re-examination tended to fall somewhere in between those of the full group of baseline participants and those of the baseline non-participants. This made them largely resemble the full background population, with an MSE of 4.4. The substitution and extrapolation approaches also resulted in distributions that were closer to those in the full background population than those observed in the full group of baseline participants, although on average not as close as when simply considering non-participants in the re-examination (MSE of 5.4 for the substitution approach and 8.4 for the extrapolation approach).

In Table 2, we report mortality and disease incidence rates across the different groups under consideration. Baseline participants experienced substantially lower all-cause mortality, CVD mortality, cancer mortality, smoking-related mortality, CVD incidence, incidence of smoking-related conditions, and incidence or alcohol-related conditions than baseline non-participants. On the other hand, cancer incidence was somewhat higher among baseline participants than among baseline non-participants.

**Table 2:** Mortality and incidences (events per 10,000 person-years)

	Background population (*n* = 65,068)	Baseline non-participants (*n* = 38,594)	Baseline participants (*n* = 26,474)	Baseline participants, participating in re-examination (*n* = 21,868)	Baseline participants, not participating in re-examination (*n* = 4,606)	Substitution approach to reconstruct background population	Extrapolation approach to reconstruct background population
All-cause mortality	284	318	237	232	260	251	276
CVD mortality	97	110	78	75	91	86	100
Cancer mortality	88	93	80	80	79	79	78
Smoking-related mortality	33	39	24	24	28	27	31
Alcohol-related mortality	2.6	3.4	1.5	1.2	2.7	2.2	3.6
CVD incidence	178	191	158	155	174	167	184
Cancer incidence	284	279	291	296	266	276	248
Smoking-related incidence	76	89	59	57	70	66	78
Alcohol-related incidence	25	32	15	13	24	20	30
MSE			380	482	114	178	167

Individuals were followed from September 2001 until death or a disease outcome under consideration, first emigration, or at the latest until December 31, 2016. Incidence refers to death or hospital visit. MSE refers to mean squared error, calculated across all mortalities and disease incidences in the table, in a comparison with the background population

Again, baseline participants who did not participate in the re-examination tended to be rather similar to the full background population, displaying much less deviation from the background population (MSE = 114) than what the full group of baseline participants did (MSE = 380). The substitution and extrapolation approaches also yielded improvements compared to the full group of baseline participants, although not on average as good as when simply zooming in on non-participants in the re-examination (MSE = 178 with the substitution approach and 167 with the extrapolation approach). Almost throughout, the results from the substitution approach were inferior to those seen when simply considering baseline participants who did not participate in the re-examination. The extrapolation approach however produced results more similar to the background population in several regards, including all-cause mortality, CVD mortality, smoking-related mortality, and smoking-related incidence. Cancer mortality was difficult to approximate regardless of method.

**Fig. 2:**
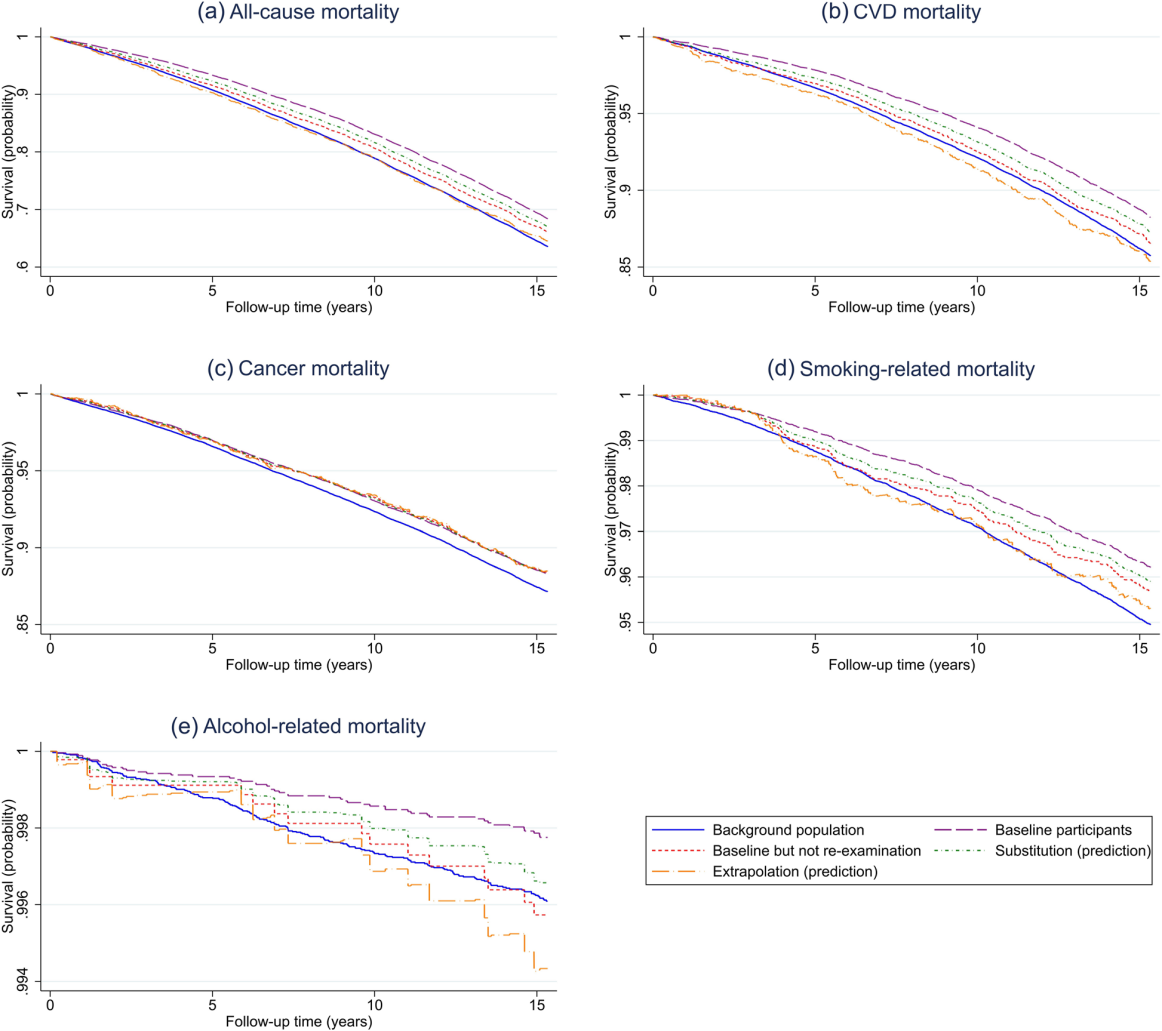
Kaplan–Meier (KM) curves for mortality, showing the cumulative probability of survival among the full background population, the baseline participants, and the baseline participants not participating in the rescreening. The curves representing the substitution and extrapolation approaches aim to predict outcomes in the full background population. CVD deaths are defined based on ICD codes I00-I99 and cancer deaths based on ICD codes C00-C99. The ICD codes used to define smoking- and alcohol-related deaths are provided in Supplementary Table S1. Note that, to enhance clarity, the range of the y-axis varies across the figures

**Fig. 3:**
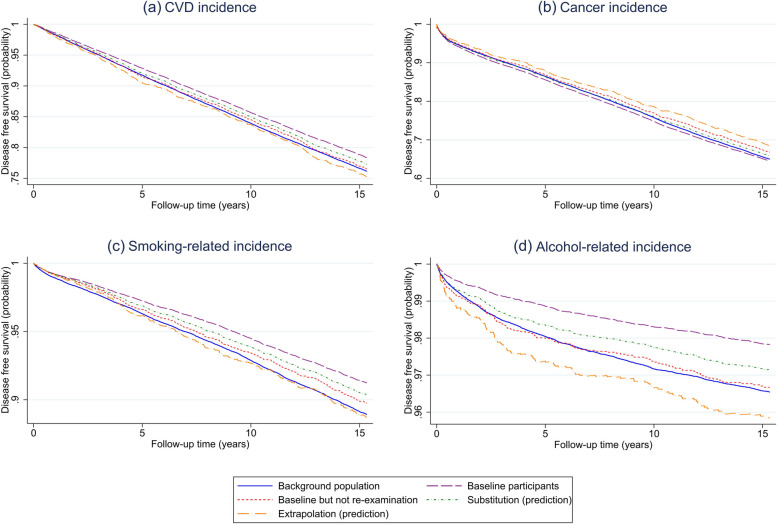
Kaplan–Meier (KM) curves for disease incidence showing the cumulative probability of disease-free survival among the full background population, the baseline participants, and the baseline participants not participating in the rescreening. The curves representing the substitution and extrapolation approaches aim to predict outcomes in the full background population. ICD codes for CVD, cancer, smoking-related, and alcohol-related events are provided in the Data section and in Supplementary Table S1. Note that, to enhance clarity, the range of the y-axis varies across the figures

Figure 2 shows KM curves for mortality and Fig. 3 for disease incidences. Each graph includes one curve for the background population, one for the full group of baseline participants, one for baseline participants who did not participate in the re-examination, one for the results based on the substitution approach, and one for the results based on the extrapolation approach. Over a 10-year follow-up, for example, the all-cause mortality risk was 21.1% in the full population but only 16.9% among baseline participants. The extrapolation approach provided an almost perfect fit, with 21.1%. Close to perfect fit over a 10-year follow-up was also obtained when applying the extrapolation method to smoking-related mortality, alcohol-related mortality, CVD incidence and smoking-related incidence. Extrapolation provided good approximations for these outcomes over the full follow-up period of approximately 15 years as well, the main exception being alcohol-related mortality. The substitution approach provided a good fit for cancer incidence, throughout the follow-up. Zooming in on baseline participants who did not participate in the re-examination provided a close to perfect fit for alcohol-related incidence.

In Table 3, we report prevalences of smoking, risky drinking, and overweight/obesity across the same groups as before, except for the full population, where these numbers are unobserved. Compared to the full group of baseline participants, those who did not participate in the re-examination displayed less healthy features, with for example 29% smoking regularly, as compared to 23% in the full group of baseline participants. The substitution approach yielded a population prevalence of 27% regular smokers, and the extrapolation approach as much as 34%. For risky drinking and body mass, results were more similar across the different approaches. Among the baseline participants, 8% were high-risk drinkers; zooming in on those not participating in re-examination or applying the substitution method yielded 9%, whereas the extrapolation model suggested 10%. Among the baseline participants, 13% were obese, a number that increased to 16% when considering baseline participants not participating in the re-examination, 15% with the substitution approach, and 17% with extrapolation.

**Table 3:** Baseline prevalences (%)

	Baseline participants (*n* = 26,474)	Baseline participants, participating in re-examination (*n* = 21,868)	Baseline participants, not participating in re-examination (*n* = 4,606)	Substitution approach to reconstruct background population	Extrapolation approach to reconstruct background population
*Smoking*	*(n* = *26,463)*	*(n* = *21,861)*	*(n* = *4,602)*		
Yes, regularly	23	22	29	27	34
Yes, occasionally	4.5	4.4	4.6	4.6	4.8
No, stopped	34	34	33	33	32
No, never	39	40	33	35	29
*Alcohol*	*(n* = *26,422)*	*(n* = *21,833)*	*(n* = *4,589)*		
High risk	8.0	7.8	9.2	8.7	10
*Body mass*	*(n* = *26,442)*	*(n* = *21,845)*	*(n* = *4,597)*		
Normal/underweight (BMI < 25)	47	48	45	46	43
Overweight (25 ≤ BMI < 30)	40	40	40	40	40
Obese (BMI ≥ 30)	13	13	16	15	17

Prevalences are obtained from the MDC baseline screening

## Discussion

Many studies have documented that participants in cohort studies tend to differ from non-participants along a range of dimensions, including socioeconomic status, sex, and health-related behaviors [1, 33–37]. Similarly, it has been shown that participants in re-examinations tend to differ from those only participating at baseline, with differences typically observed along the same dimensions and in the same directions as when comparing baseline participants with non-participants [29, 38–43]. Drawing on the concept of a continuum of resistance, we here proposed that more accurate estimates of outcomes and characteristics in a background population may be obtained by exploiting data on baseline participants who did not participate in a follow-up examination.

Using data from the MDC study, we examined how well baseline participants who did not participate in a re-examination 5-year after baseline resembled the participants and the full invited population with respect to background characteristics and disease history, and outcomes including mortality and incidence of disease. Moreover, we applied two methods from the continuum of resistance literature, aiming to determine the ability of these methods to approximate the distributions of outcomes and characteristics in the full invited population. We focused on disease outcomes and background characteristics as such rather than associations between different outcomes and characteristics, as our previous examinations of the MDC study [7] as well as evidence from other cohorts of self-selected participants [27, 28, 35, 44–47] suggest that associations observed in participant samples tend to be similar to those in the full populations even without adjustments, at least when measured on a relative scale. However, outcomes such as mortality have been found to differ markedly across MDC participants and the full population even after standard adjustments [7, 30], implying that novel approaches to improve generalizability of such outcomes are needed.

The two estimation methods were relatively successful in reproducing the distributions of characteristics and outcomes in the full population. Particularly for mortality, the extrapolation method tended to provide a good fit. On average across all outcomes and characteristics, the best fit was however provided by the most straightforward approach: simply considering the unadjusted group of baseline participants who did not participate in the re-examination.

While no approach produced more accurate results than another throughout, the results from the different approaches often provided plausible ranges for the outcomes and characteristics that were observable in the background population. If assuming that similar patterns would hold for health-related behaviors and anthropometrics, which were unobservable in the full background population, we can conclude that the participant sample of the MDC study appears to underestimate the prevalence of regular smoking, perhaps by somewhere in between four and eleven percentage points. The prevalence of risky drinking also appears to be underestimated, perhaps by one to two percentage points, as does the prevalence of obesity, perhaps by two to four percentage points. Whether this is truly the case is impossible to know. However, the success of the extrapolation approach to reproduce smoking-related mortality and disease incidence in the background population and the success of the unadjusted participants to approximate alcohol-related mortality and incidence gives an indication the same methods might be reliable also in estimating population prevalences of smoking and risky drinking. Hence, particularly smoking prevalence may have been substantially underestimated in the MDC study.

In one previous study, authors compared characteristics and behaviors of MDC participants with those of similar-aged participants in a mailed health survey that was conducted in Malmö around the same time as the MDC study, but which had a substantially higher response rate [30]. The prevalence of current smoking in the survey was only two percentage points higher than the numbers observed in the MDC study, and the prevalence of obesity was even three percentage points lower. Furthermore, the prevalence of drinking during the past year (a variable that we lack access to) was two percentage points lower in the health survey than in the MDC study. These numbers clearly contrast with ours. However, while participation in this survey was much higher than in the MDC study (75% vs 40%), a fair amount of the invited population did nevertheless not respond, meaning that the survey may as well have been non-representative. As has been pointed out [48], studies with higher participation rates do not necessarily produce more generalizable results than those with lower ones, and in a review of 235 estimates from 30 different surveys, authors found only a moderate correlation between nonresponse rates and lack of generalizability [49]. Bias due to selective participation largely depends on the strength of the association between sample participation and the outcome or association of interest [49, 50], and a stronger association between the two can outweigh the benefit of a higher participation rate.

In this study, we found that a relatively small group – those 7% of the population who participated at baseline but not in the re-examination – was rather representative of the full population. The finding points to the importance of not equating representativity with the relative size of the group from which results are obtained and draws attention to the potential usefulness of exploiting data from subgroups of participants with a smaller participation propensity. While our study exploited data from a 5-year re-examination, meaning that results could not have been obtained within 5 years after baseline, future studies based on other cohorts may exploit participation in re-examinations that take place sooner after baseline, or examinations that take place in parallel or even before the baseline screening. By zooming in on baseline participants who did not participate in other examinations or by applying the methods from the continuum of resistance literature that we have described, researchers may increase the likelihood of obtaining generalizable results. At a minimum, results from such investigations should serve as a useful sensitivity check when studying a cohort of self-selected individuals.

## Conclusions

Prevalences, incidences, and other quantities obtained for baseline participants in a cohort study may not generalize to the full population of invitees. As shown in this article, however, substantial improvements may be obtained by exploiting data on whether baseline participants also participated in a re-examination, a finding that presumably reflects that participation in the re-examination depends on factors similar to those determining participation at baseline. Our approach can be applied to any cohort study where these is information on whether baseline participants also participated in another examination, potentially allowing for improved generalizability across a wide variety of settings.

### Supplementary Information

**Additional file 1: Table S1.** ICD codes for smoking- and alcohol-related outcomes.

## Data Availability

The database used in this study is closed but researchers with an ethical approval from the Swedish Ethical Review Authority may contact the first author A.N. to gain access. We received access to the data via the register holders (the Malmö Diet and Cancer study at Lund University, Statistics Sweden, and the National Board of Health and Welfare in Sweden) after an ethical approval by the Regional Ethics Review Board in Lund.

## Abbreviation

MDC: Malmö Diet and Cancer
