# Supplementary material for: Can non-participants in a follow-up be used to draw conclusions about incidences and prevalences in the full population invited at baseline? An investigation based on the Swedish MDC cohort
Source: BMC Med Res Methodol. 2023 Oct 11;23:228. doi: 10.1186/s12874-023-02053-w (PMC10568880; doi:10.1186/s12874-023-02053-w)
Supplement: Supplementary file 1 — Additional file 1: Table S1. ICD codes for smoking- and alcohol-related outcomes. [file 12874_2023_2053_MOESM1_ESM.docx]

**Table S1** ICD codes for smoking- and alcohol-related outcomes

| Panel A: Smoking-related conditions |  |
| --- | --- |
| C33 | Malignant neoplasm of trachea |
| C34 | Malignant neoplasm of bronchus and lung |
| J41-44 | Chronic obstructive pulmonary disease |
| Panel B: Alcohol-related conditions |  |
| E244 | Alcohol-induced pseudo-Cushing's syndrome |
| F10 | Mental and behavioral disorders due to use of alcohol |
| G312 | Degeneration of nervous system due to alcohol |
| G621 | Alcoholic polyneuropathy |
| G721 | Alcoholic myopathy |
| I426 | Alcoholic cardiomyopathy |
| K292 | Alcoholic gastritis |
| K70 | Alcoholic liver disease |
| K852 | Alcohol induced acute pancreatitis |
| K860 | Alcohol-induced chronic pancreatitis |
| O354 | Maternal care for (suspected) damage to fetus from alcohol |
| T510 | Alcohol use, unspecified with intoxication, unspecified |
